# Abundant and Rare Bacterial Taxa Structuring Differently in Sediment and Water in Thermokarst Lakes in the Yellow River Source Area, Qinghai-Tibet Plateau

**DOI:** 10.3389/fmicb.2022.774514

**Published:** 2022-03-29

**Authors:** Ze Ren, Cheng Zhang, Xia Li, Kang Ma, Baoshan Cui

**Affiliations:** ^1^Advanced Institute of Natural Sciences, Beijing Normal University, Zhuhai, China; ^2^School of Environment, Beijing Normal University, Beijing, China; ^3^School of Engineering Technology, Beijing Normal University, Zhuhai, China

**Keywords:** thermokarst lakes, Qinghai-Tibet Plateau, species turnover, nestedness, abundant and rare taxa, 16S

## Abstract

Thermokarst lakes are forming from permafrost thaw and are severely affected by accelerating climate change. Sediment and water in these lakes are distinct habitats but closely connected. However, our understanding of the differences and linkages between sediment and water in thermokarst lakes remains largely unknown, especially from the perspective of community assembly mechanisms. Here, we examined bacterial communities in sediment and water in thermokarst lakes in the Yellow River Source area, Qinghai-Tibet Plateau. Bacterial taxa were divided into abundant and rare according to their relative abundance, and the Sorensen dissimilarity (β_*sor*_) was partitioned into turnover (β_*turn*_) and nestedness (β_*nest*_). The whole bacterial communities and the abundant and rare subcommunities differed substantially between sediment and water in taxonomical composition, α-diversity, and β-diversity. Sediment had significantly lower α-diversity indexes but higher β-diversity than water. In general, bacterial communities are predominantly governed by strong turnover processes (β_*turn*_/β_*sor*_ ratio of 0.925). Bacterial communities in sediment had a significantly higher β_*turn*_/β_*sor*_ ratio than in water. Abundant subcommunities were significantly lower in the β_*turn*_/β_*sor*_ ratio compared with rare subcommunities. The results suggest that the bacterial communities of thermokarst lakes, especially rare subcommunities or particularly in sediment, might be strongly structured by heterogeneity in the source material, environmental filtering, and geographical isolation, leading to compositionally distinct communities. This integral study increased our current knowledge of thermokarst lakes, enhancing our understanding of the community assembly rules and ecosystem structures and processes of these rapidly changing and vulnerable ecosystems.

## Introduction

Permafrost is widespread in high latitude and high elevation regions, covering approximately one-quarter of the land surface in the Northern Hemisphere and experiencing more severe warming than the remainder of the globe (Qiu and Cheng, 1995; Zhang et al., 1999). Thermokarst lakes are forming as a result of permafrost thaw, acting as important and widespread aquatic ecosystems in cold regions (Kokelj and Jorgenson, 2013; Farquharson et al., 2016) with significant roles in hydrological, ecological, and biogeochemical processes (Chin et al., 2016; In’T Zandt et al., 2020; Manasypov et al., 2021). In the Arctic and sub-Arctic area, thermokarst lakes cover up to 40% of the permafrost area (de Jong et al., 2018). As an indicator of permafrost degradation, thermokarst lakes are suffering substantial changes in size and abundance owing to accelerating permafrost thaw (Karlsson et al., 2012; Pastick et al., 2019). Under accelerating climate change, the evolution process of thermokarst lakes, including expansion, erosion, shrinkage, and disappearance, will be accelerated (Vincent et al., 2013; Luo et al., 2015; Biskaborn et al., 2019), resulting in significant impacts on regional environmental security and global biogeochemical processes (Luo et al., 2015). However, we lack knowledge in the ecosystem structure, function, and processes of thermokarst lakes compared with temperate lakes.

In lake ecosystems, bacteria play pivotal roles in ecosystem structuring and functioning. Bacterial communities exhibit high compositional and functional diversities and variabilities, with a relatively few abundant taxa coexisting with a considerable proportion of rare taxa (Lynch and Neufeld, 2015). In these enormously complex bacterial communities, abundant and rare taxa have fundamentally different characteristics and ecological roles than abundant taxa (Logares et al., 2014). For example, abundant taxa contribute predominantly to biomass production and energy flow, whereas rare taxa contribute mostly to species richness and redundant functions (Pedros-Alio, 2012; Debroas et al., 2015). More and more studies have been conducted to unravel the differences between abundant and rare subcommunities in various environments, such as coastal and marine environments (Campbell et al., 2011; Logares et al., 2014), inland waters (Liu et al., 2015; Xue et al., 2018; Ren et al., 2020), and soil (Jiao and Lu, 2019; Xue et al., 2020). These studies indicated that abundant and rare subcommunities present contrasting community patterns and processes and are subject to distinct environmental factors. Assessment of bacterial communities by considering abundant and rare subcommunities is also crucial for understanding the heterogeneous and fast-changing nature of thermokarst lake ecosystems.

In lake ecosystems, sediment and water are two distinct but closely interconnected environments (Carter et al., 2003; Parker et al., 2016). For example, these two environments have different chemical and physical properties but interact intimately through materials deposition from water to sediment and resuspension from sediment to water (Roeske et al., 2012). These two habitats host different assemblages of microorganisms with tremendous diversity, which play vital roles in maintaining and driving ecosystem structure and processes (Lozupone and Knight, 2007; Roeske et al., 2012; Ren et al., 2019). Moreover, the variations of bacterial communities in sediment and water are driven differently by a variety of factors (Gasol et al., 2002; Simek et al., 2008; Ren et al., 2019, 2021a). For thermokarst lakes, their formation (permafrost thaw) and evolution (horizontal and vertical permafrost degradation) mechanisms suggest that sediment and water have very close relationships in thermokarst lakes. Thermokarst processes stimulate the release of carbon, nutrients, and even heavy metals from deeper permafrost (sediment) to water, and these releasing processes can be further accelerated by microbial activities and climate change (Bowden, 2010; In’T Zandt et al., 2020; Manasypov et al., 2021). Intensifying climate change is beginning to unlock more materials and microorganisms from sediment to water (Graham et al., 2012; Mackelprang et al., 2017). In cold regions, thermokarst lakes are especially important due to their abundance (Polishchuk et al., 2017), massive storage of water, carbon, and nutrients (Reyes and Lougheed, 2015), as well as their enormous contributions of greenhouse gases (Walter et al., 2006; Serikova et al., 2019; In’T Zandt et al., 2020). Although immense amounts of microbial research have been conducted in thermokarst lakes, the overwhelming majority of which focused on surface water (Tranvik, 1989; Shirokova et al., 2013; Vucic et al., 2020) rather than sediment or both. However, the differences and linkages between sediment and water in thermokarst lakes remain largely unknown.

In addition, it has been recently studied that there are close relationships between community assembly processes and biogeochemical function (Bier et al., 2015; Graham et al., 2016a; Graham and Stegen, 2017; Xun et al., 2019; Le Moigne et al., 2020). There is much evidence to support that species richness and abundance are important determinants of ecosystem functions (Wardle et al., 2011; Xun et al., 2019). Assembly processes (related to β-diversity), including deterministic (e.g., selection) and stochastic (e.g., dispersal) processes, inevitably shift community diversity and composition, therefore, resulting in downstream impacts on ecosystem functions (Graham et al., 2016a; Leibold et al., 2017; Mori et al., 2018). For example, Luan et al. (2020) and Le Moigne et al. (2020) suggested that stochastic dispersal suppresses the metabolism and mineralization of organic carbon in soil and water, respectively. Given the long-term goal of understanding how microbial community variations affect the biogeochemistry of thermokarst lakes, it is necessary to consider the implications of microbial community assembly on biogeochemical function. Revealing the bacterial community structuring patterns of abundant and rare taxa in sediment and water in thermokarst lakes can improve our understanding of biogeochemical processes in these lakes under accelerating climate change.

As the third pole of the world, up to 40% of the Qinghai-Tibet Plateau is covered by permafrost and is highly sensitive to climate change (Zou et al., 2017). The ongoing global warming has accelerated permafrost degradation, resulting in extensive changes of thermokarst lakes with lake number and lake area increasing (Luo et al., 2015; Zhang et al., 2017). In this study, we investigated the bacterial communities in sediment and water in thermokarst lakes of the Yellow River Source area. Our object was to reveal (1) the assemblage structure processes of abundant and rare taxa in sediment and water, (2) the responses of abundant and rare subcommunities to environmental variables, and (3) the differences and linkages between sediment and water. The integral understanding of bacterial community in both sediment and water could provide insights into community assembly rules and ecosystem structures and processes of the thermokarst lakes.

## Materials and Methods

### Study Area, Field Sampling, and Chemical Analysis

**FIGURE 1:**
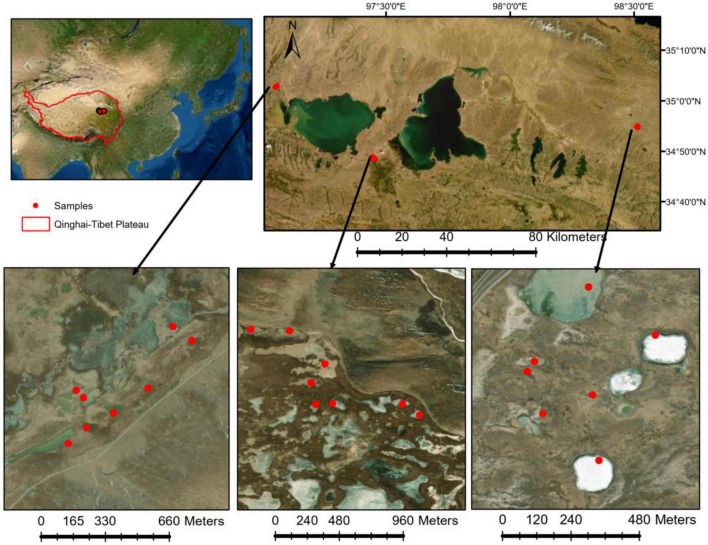
Water and sediment samples were collected from 23 lakes in early July 2020 in Yellow River Source area on Qinghai-Tibet Plateau.

This study was conducted in the Yellow River Source area on the Qinghai-Tibet Plateau (Figure 1). In early July 2020, we sampled 23 thermokarst lakes in the study area. The elevation of the studied lake surface ranged from 4,200 to 4,350 m above sea level. In each lake, both water samples and sediment samples were collected. The conductivity and pH of the lake water were measured *in situ* using a multiparameter instrument (YSI ProPlus, Yellow Springs, Ohio). Because the thermokarst lakes are very shallow, only surface water samples were collected at a depth of 0.3–0.5 m. In each lake, three 1-L water samples were filled in acid clean bottles and transported to the laboratory for chemical analyses, including dissolved organic carbon, total nitrogen (TN), and total phosphorus (TP). Microbial samples were collected by filtering 200-mL water onto a 0.2-μm polycarbonate membrane filter (Whatman, United Kingdom) from each of the three 1-L water samples, respectively. For each lake, three filters were combined into one composite sample and frozen in liquid nitrogen immediately in the field and stored at –80°C in the lab until DNA extraction. Sediment samples were collected using a Ponar Grab sampler. The top 5 cm of the sediment was collected and homogenized. Sediment microbial samples were connected in a 45-ml sterile centrifuge tube, frozen in liquid nitrogen in the field, and stored at –80°C in the lab for DNA extraction. The remaining sediments were air-dried for the determination of chemical properties, including pH, conductivity, sediment organic carbon, TN, and TP. The basic chemical properties of sediment and water samples are summarized in Supplementary Table 1.

### DNA Extraction, Polymerase Chain Reaction, and Sequencing

The water and sediment microbial samples were used to extract genomic DNA using the DNeasy PowerSoil Kit (QIAGEN, Germany) following the manufacturer’s protocols. The genome DNA was used as the template for polymerase chain reaction amplification with the barcoded primers and Tks Gflex DNA Polymerase (Takara, United States). The hypervariable V3–V4 regions of bacterial 16S ribosomal RNA were amplified using universal primers 343F 5′-TACGGRAGGCAGCAG-3′ and 798R 5′-AGGGTATCTAATCCT-3′ (Nossa et al., 2010). To minimize amplification bias, three individual polymerase chain reaction amplifications were performed using the following procedure: initial denaturation at 94°C for 5 min, 24 cycles of denaturation at 94°C for 30 s followed by annealing at 56°C for 30 s and extension at 72°C for 20 s, and final extension step at 72°C for 5 min. Amplified DNA was verified by agarose gel electrophoresis, purified using the AMPure XP beads (Beckman, United States), and quantified using Qubit dsDNA assay kit (Thermo Fisher Scientific, United States). Sequencing of the amplicon libraries was conducted on an Illumina MiSeq platform (Illumina, San Diego, CA, United States) according to the manufacturer’s instructions. Raw sequence data can be accessed at the China National Center for Bioinformation (CRA004269 under the project PRJCA005279).

## Data Analyses

Raw sequence data were preprocessed using Trimmomatic software (version 0.35) (Bolger et al., 2014) to detect and cut off ambiguous bases and low-quality sequences with an average quality score below 20. After trimming, paired-end reads were assembled using FLASH software (Reyon et al., 2012). Parameters of assembly were as follows: 10 bp of minimal overlapping, 200 bp of maximum overlapping, and 20% of maximum mismatch rate. Sequences were performed further denoising using QIIME 1.9.1 (Caporaso et al., 2010) as follows: reads with ambiguous, homologous sequences or below 200 bp were abandoned; reads with 75% of bases above Q20 were retained; reads with chimera were detected and removed. Clean reads were subjected to primer sequences removal and clustering to generate operational taxonomic units (OTUs) against the SILVA 132 database (Quast et al., 2013) using QIIME. To avoid the bias of surveying efforts, the sequence data were normalized at a depth of 27,890 sequences per sample (Supplementary Figure 1). After quality filtering and the removal of chimeric sequences, 1,282,940 high-quality sequences were acquired and clustered into 27,889 OTUs at 97% nucleotide similarity level in sediment and water of the studied thermokarst lakes.

Bacterial taxa were defined as abundant and rare according to their relative abundance. In this study, OTUs with a relative abundance ≥ 0.1% of the total sequences were defined as abundant, and OTUs with a relative abundance < 0.01% were defined as rare. The α-diversity indices, including Chao 1, observed OTUs, Shanon, and phylogenetic diversity (PD whole tree), were calculated using QIIME 1.9.1 (Caporaso et al., 2010). β-Diversity is a central concept in ecology and biogeography to assess the compositional variations among species communities (Whittaker, 1972). Understanding patterns and processes of β-diversity allows connecting spatiotemporal community structure to ecological processes (Legendre and De Caceres, 2013; Socolar et al., 2016). β-Diversity can be further partitioned into two subcomponents to measure the spatial turnover of species and the nestedness of assemblages (Baselga, 2010; Legendre, 2014). Species spatial turnover reflects the gains and losses of species between sites due to environmental sorting and/or constraints of geographic barrier (Gaston et al., 2007a; Baselga, 2010). In contrast, nestedness implies that the species at a depauperate site is a subset of assemblages of a species-rich site (Leprieur et al., 2011; Baselga, 2012). Partitioning β-diversity into the components of turnover and nestedness is essential for understanding the mechanisms shaping biodiversity patterns across various spatial and temporal scales (Baselga, 2010). Thus, to further reveal the mechanisms underlying the discrepancies observed between the abundant and rare subcommunities, as well as between bacterial communities in sediment and water, the Sorensen dissimilarity (β_*sor*_, a β-diversity metric that measures compositional differences between sites independent of richness) was partitioned to a turnover component (β_*turn*_) and a nestedness-resultant fraction (β_*nest*_) (Baselga, 2010) using the “betapart 1.5.4” package (Baselga and Orme, 2012). To estimate the habitat niche occupied by each species, we calculated Levin’s niche width (Levins, 1968) using the “spaa” package (Zhang, 2016) in R. The formula is $B_{i}=1/\sum_{1}^{n}p_{i}^{2}$. B_*i*_ represents the niche width of OTU_*i*_ across the communities, n is the total number of communities, and p_*i*_ is the proportion of OTU_*i*_ in each community. The species with a higher niche width distributed more evenly along a wider habitat range than those with a lower niche width. The differences of the whole, abundant, and rare communities/subcommunities between sediment and water were revealed by principal coordinates analysis based on Bray–Curtis distance and tested by analysis of variance using distance matrices, analysis of similarity, and multi-response permutation procedure analysis using “vegan 2.5-7” package (Oksanen et al., 2007). Structural equation model was conducted using the “piecewiseSEM 2.1.2” package (Lefcheck, 2016) to quantify the effects of environmental variables on β-diversity of bacterial communities in sediment and water, as well as the biological and physicochemical relationships between sediment and water. All the analyses were conducted in R 4.0.4 (R Core Team, 2017).

## Results

### Bacterial Communities in Sediment and Water

**FIGURE 2:**
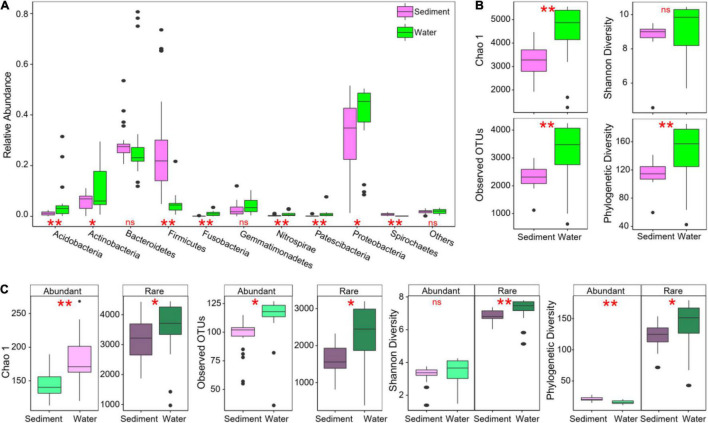
Bacterial community composition and alpha diver in sediment and water of studied thermokarst lakes. **(A)** Differences in taxonomic composition of overall communities between sediment and water. **(B)** Differences in α-diversity of overall communities between sediment and water. **(C)** Differences in α-diversity between abundant and rare subcommunities. Differences were assessed using Wilcoxon rank-sum test with “ns”, “*”, and “**” representing non-significance, *P* < 0.05, and *P* < 0.01, respectively.

In total, 17,178 OTUs were detected in sediment, and 17,455 OTUs were detected in water. For the assigned OTUs in sediment, Proteobacteria (30%), Bacteroidetes (29%), Firmicutes (28%), and Actinobacteria (6%) were the dominant phyla (with an average relative abundance > 5%, Figure 2A). In water bacterial communities, Proteobacteria (40%), Bacteroidetes (30%), Actinobacteria (11%), and Acidobacteria (5%) were the dominant phyla (Figure 2A). In the top 10 most abundant phyla in these thermokarst lakes, sediment had significantly higher Firmicutes and Spirochaetes, whereas lower Acidobacteria, Actinobacteria, Fusobacteria, Nitrospirae, Patescibacteria, and Proteobacteria than water (Figure 2A). The top 25 OTUs in sediment and water were significantly different (Supplementary Figure 4). The most abundant OTU in sediment belonged to the genus *Escherichia-Shigella*. The most abundant OTU in water belonged to the genus *Candidatus Aquiluna.* Moreover, bacterial α-diversity was significantly lower in sediment than in water in Chao 1, observed OTUs, and phylogenetic diversity (Figure 2B).

**FIGURE 3:**
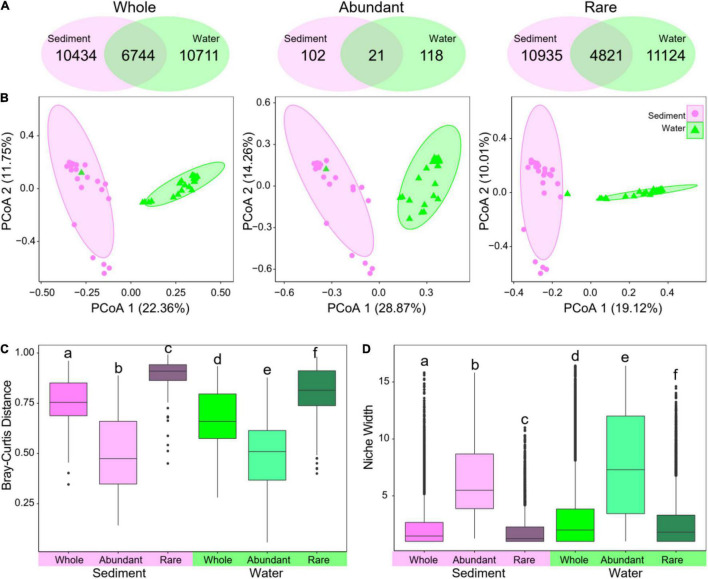
Structural differences of bacterial communities between sediment and water in whole, abundant, and rare subcommunities. **(A)** Venn diagram showing unique and shared OTUs in sediment and water. **(B)** Principal coordinates analysis (PCoA) based on Bray–Curtis distance using relative abundance of OTUs. **(C)** β-diversity measured using Bray–Curtis distance. **(D)** Niche width of OTUs. Different low-case letters in **(C,D)** represent significant differences assessed using analysis of variance.

Sediment and water harbored a large amount of unique OTUs, which were only presented in sediment (*n* = 10,434) or water (*n* = 10,711), whereas 6,744 OTUs were detected in both (Figure 3A). Substantial differences were observed between sediment and water bacterial communities based on the principal coordinates analysis results (Figure 3B). These results were further confirmed by the results of analysis of variance using distance matrices, analysis of similarity, and multi-response permutation procedure analysis analyses (Supplementary Table 2). The sediment bacterial communities had a significantly higher β-diversity (Figure 3C) but lower niche width (Figure 3D) than water ones. The results indicate that the sediment and water environments harbored distinct bacterial communities in taxonomic composition as well as α- and β-diversity.

### Abundant and Rare Subcommunities in Sediment and Water

In the whole bacterial communities, a large proportion of the OTUs was identified as rare taxa (69.6% for sediments and 69.8% for water in the number of OTUs), but rare taxa only accounted for 25.1 and 22.3% of relative abundance in sediments and water (Supplementary Figure 2), respectively. Conversely, a very small proportion of the OTUs was identified as abundant taxa (4.2 and 3.9% in both sediments and water, respectively), which account for 39.8 and 40.7% of the average relative abundance in each sample (Supplementary Figure 2). For both abundant and rare subcommunities, α-diversity was also significantly lower in sediment than in water (Figure 2C). Venn diagram showed that only 21 abundant and 4,821 rare OTUs were shared in both sediment and water environments (Figure 3A).

Moreover, abundant and rare subcommunities also had distinct structures between sediment and water (Figure 3B). When comparing abundant and rare subcommunities, abundant subcommunities had a significantly lower β-diversity (Bray–Curtis distance) but higher niche width than rare subcommunities in both sediment and water (Figures 3C,D). When comparing sediment and water, abundant subcommunities had a lower β-diversity and niche width in sediment than in water, whereas rare subcommunities had a higher β-diversity but lower niche width in sediment than in water (Figures 3C,D). The results indicate distinct distribution patterns and taxonomic composition between abundant and rare subcommunities in sediment and water.

### Turnover and Nestedness

**FIGURE 4:**
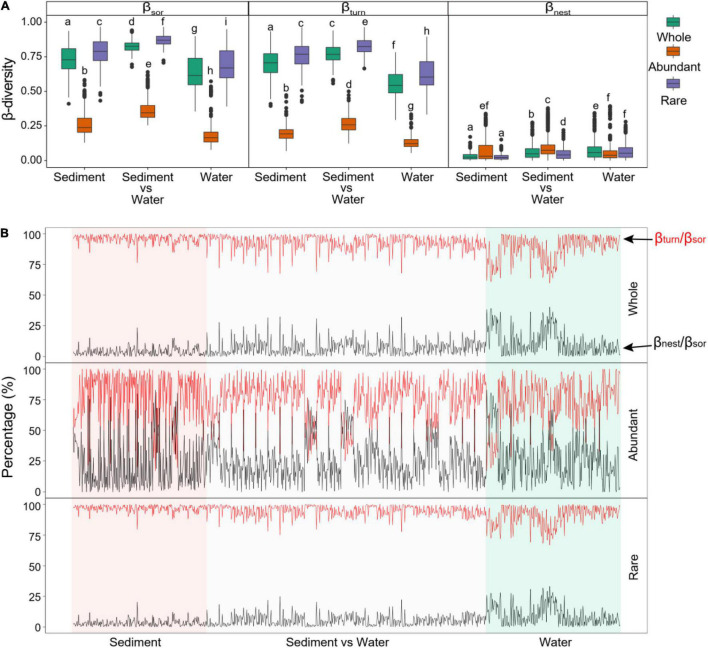
β-Diversity partitioning results between pairs of sites in only sediment samples, between sediment and water samples (sediment *vs.* water), and only water samples. **(A)** Differences in total β-diversity calculated as Sorensen dissimilarity (β_*sor*_), turnover component (β_*turn*_), and nestedness-resultant fraction (β_*nest*_) between whole, abundant, and rare assemblages. Different low-case letters represent significant differences assessed using analysis of variance. **(B)** Contributions of turnover component to total Sorensen dissimilarity (β_*turn*_/β_*sor*_ ratio) and of nestedness-resultant fraction to total Sorensen dissimilarity (β_*nest*_/β_*sor*_ ratio) for paired sites.

Based on β-partitioning, the Sorensen dissimilarity (β_*sor*_, a β-diversity metric that measures compositional differences between sites) was partitioned into a turnover component (β_*turn*_) and a nestedness-resultant fraction (β_*nest*_) (Figure 4A). The estimated β_*sor*_ values of the whole bacterial communities between paired sites were 0.733 for only sediment samples, 0.827 for sediment vs. water samples, and 0.638 for only water samples (Figure 4A). The estimated β_*sor*_ values of abundant subcommunities between paired sites were 0.273 for only sediment samples, 0.365 for sediment *vs.* water samples, and 0.198 for only water samples (Figure 4A). The estimated β_*sor*_ values of rare subcommunities were 0.786 for only sediment samples, 0.871 for sediment *vs.* water samples, and 0.691 for only water samples (Figure 4A). Abundant subcommunities had a significantly lower β_*sor*_ than rare subcommunities (Figure 4A). Sediment communities had a significantly higher β_*sor*_ than water communities (Figure 4A).

Moreover, the estimated β_*sor*_ of bacterial communities was mainly contributed by the turnover component with an average β_*turn*_/β_*sor*_ ratio of 0.925 for the whole communities (Figure 4B). Abundant subcommunities had a significantly lower contribution of β_*turn*_ to β_*sor*_ (β_*turn*_/β_*sor*_ ratio of 0.746) than that of rare subcommunities (β_*turn*_/β_*sor*_ ratio of 0.942) (Figure 4B and Supplementary Figure 3). Comparing different habitats, sediment had a significantly higher β_*turn*_/β_*sor*_ ratio than water (Figure 4B and Supplementary Figure 3).

### Environmental Responses of Abundant and Rare Subcommunities in Sediment and Water

**FIGURE 5:**
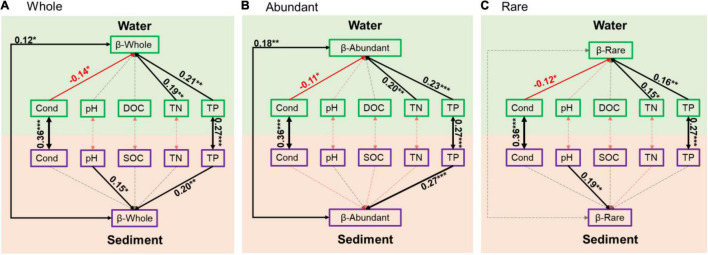
Structural equation model illustrating relationships between variation of environmental variables and β-diversity of bacterial communities in sediment and water in **(A)** whole community, **(B)** abundant subcommunities, and **(C)** rare subcommunities. Solid and dashed arrows represent significant and non-significant relationships, respectively. Red and black arrows represent negative and positive relationships, respectively. Significant path coefficients were shown adjacent to path with *, **, and *** that denote the significant level of *p* < 0.05, *p* < 0.01, and *p* < 0.001, respectively.

Based on the structural equation model results, sediment and water had close associations in conductivity and TP (Figure 5). In addition, sediment and water had close associations in the β-diversity of the whole bacterial communities and the abundant subcommunities (Figure 5). In sediment, pH and TP had positive effects on the β-diversity of the whole communities, TP had positive effects on β-diversity of the abundant subcommunities, and pH had positive effects on β-diversity of the rare subcommunities (Figure 5). In water, however, conductivity had negative effects, and TN and TP had positive effects on the β-diversity of the whole communities and the abundant and rare subcommunities (Figure 5).

## Discussion

### Differences and Linkages Between Sediment and Water

This study showed that the whole bacterial communities, as well as the abundant and rare subcommunities, differed substantially between sediment and water in taxonomical composition, α-diversity, and β-diversity. It is unequivocal that different habitats usually harbor distinct microbial assemblages (Fierer et al., 2012; Hugerth et al., 2015; Louca et al., 2016). Previous studies have demonstrated that, in lake ecosystems, sediment and water host different bacterial communities (Gough and Stahl, 2011; Yang et al., 2016; Ren et al., 2019). In our study, 8 of the 10 most abundant phylum presented significantly different relative abundance between sediment and water (Figure 2A). Specifically, Firmicutes had a mean relative abundance of 28% in sediment but only 5% in water, and an overwhelming majority of the Firmicutes OTUs were affiliated to Clostridia class, which are typically anaerobic and can dominate the bacterial communities during the decomposition process in lake sediments (Xing et al., 2011; Zhao et al., 2017). Moreover, only a small proportion of the OTUs (24.2%) was detected in both sediment and water, whereas a high proportion of the OTUs was only detected in sediment (37.4%) and water (38.4%). This result first suggests that some of the OTUs (the shared OTUs) in sediment or water had the same source from the permafrost. On the other hand, the high proportion of unique OTUs suggests strong environmental filtering and colonization of bacteria from different sources, such as atmospheric bacteria, plant-associated bacteria, and migratory birds and animals introduced bacteria.

In general, sediments usually have higher species-level diversity than water, especially in temperate lakes (Lozupone and Knight, 2007; Ren et al., 2019). In our study, however, sediment had significantly lower α-diversity than water (Figure 2B), suggesting that the water column provided more niches for bacterial taxa than sediment. This was further supported that the bacterial taxa had higher niche widths in water than in sediment (Figure 3D). Another interpretation for the lower α-diversity in sediment than in water is that the sediment had more constraints, particularly high pH, than the water column, limiting the number of taxa that could live there.

In addition to the differences, sediment and water also had close biological and physicochemical relationships (Roeske et al., 2012; Parker et al., 2016). In our study, sediment and water were closely related in conductivity and TP (Figure 5), suggesting the possible determinant of sediment on water physicochemical environments, especially on conductivity and TP (Dahl et al., 1993; Nowlin et al., 2005; Fries, 2007; Horppila, 2019). Moreover, the β-diversity, especially the β-diversity of abundant subcommunities, also had a significant relationship between sediment and water (Figure 5), suggesting the possibility that a considerable proportion of abundant taxa in sediment and water had the same source from the permafrost and the source heterogeneity might constrain the assembly processes. Thus, these connections between sediment and water might be the results of thermokarst lakes formation and evolution processes, during which nutrients and other elements are continuously released to water from permafrost and sediment (Mackelprang et al., 2017; In’T Zandt et al., 2020; Manasypov et al., 2021).

### β-Diversity and Its Different Components

In aquatic ecosystems, bacterial communities are controlled differently by various factors and processes in different environments (Gasol et al., 2002; Simek et al., 2008; Ren et al., 2019). Understanding the factors that control bacterial community variations is a central theme in ecology (Fierer and Jackson, 2006; Pla-Rabes et al., 2011). In our study, the variations of bacterial communities had close relationships with pH and TP in sediment while with conductivity, TN, and TP in water (Figure 5). Moreover, the abundant and rare subcommunities respond differently to environmental variables in sediment that abundant subcommunities only had a close relationship with TP, whereas rare subcommunities only had a close relationship with pH (Figure 5). The availabilities of key nutrients (N and P) have long been demonstrated to be essential in structuring bacterial communities (Torsvik et al., 2002; Lee et al., 2017). Nutrient dynamics usually have close interactions with bacterial communities in water and sediment in lake ecosystems (Lee et al., 2017; Ren et al., 2019). The different responses of microorganisms to nutrients root in their metabolic features and ecological strategies, as well as environmental properties (Carbonero et al., 2014). Salinity is typically one of the major environmental determinants of the microbial community in aquatic environments (Lozupone and Knight, 2007). In thermokarst lakes, sediment was formed from permafrost soil. Many studies have demonstrated that soil bacterial communities are strongly affected by pH (Fierer et al., 2012; Ren et al., 2021b).

Unraveling the underlying mechanisms driving species distribution patterns is a vital issue in ecology and biogeography (Podani and Schmera, 2016). As a key term for assessing spatial and temporal variations of microbial assembly, β-diversity can be decomposed into two distinct components: turnover and nestedness (Baselga, 2010; Legendre, 2014), which may reflect the relative importance of different underlying mechanisms in structuring communities, which varies with the spatiotemporal scales (Podani and Schmera, 2016; Viana et al., 2016). In our study, bacterial communities are predominantly governed by strong turnover processes (β_*turn*_/β_*sor*_ ratio of 0.925 for the whole communities). However, abundant subcommunities were significantly lower in the β_*turn*_/β_*sor*_ ratio compared with rare subcommunities (Figure 4B and Supplementary Figure 3). Moreover, bacterial communities in sediment had a significantly higher β_*turn*_/β_*sor*_ ratio than in water (Figure 4B and Supplementary Figure 3). Species turnover refers to species experiencing replacement of each other (gains and losses) along ecological gradients as a consequence of spatiotemporal constraints and/or environmental sorting (Leprieur et al., 2011). Therefore, we could expect that regions that have high species turnover would also possess great heterogeneities in contemporary environmental conditions and/or strong geographical isolation caused by dispersal barriers (Gaston et al., 2007a,b). In our studied thermokarst lakes, the environmental factors had high variations in both sediment and water (Supplementary Table 1). However, sediment is more isolated than water between lakes, even for the intimate neighbors in the space. Thus, it is expected that sediment had a higher β_*turn*_/β_*sor*_ ratio than water. Moreover, the ecological tolerance and niche breadth of taxa are also determinative factors for turnover rate (Leprieur et al., 2011), which is the potential reason that abundant taxa had a lower β_*turn*_/β_*sor*_ ratio than rare taxa because of higher niche width and ecological tolerance of abundant taxa. In contrast to turnover, nestedness implies another pattern of richness difference that the species at a depauperate site is a subset of assemblages of a species-rich site (Leprieur et al., 2011; Baselga, 2012). The nestedness richness difference (species loss or gain) may result from various ecological processes, such as nestedness of habitats, selective colonization and/or extinction, and interspecific variation of environmental tolerance (Whittaker and Fernandez-Palacios, 2007; Baselga, 2010). Given that the permafrost is one of the major source materials of bacterial communities in thermokarst lakes, the heterogeneity of source material would be an important constraint on the assembly processes, which need to be further studied by collecting ambient soil samples around the lake. All in all, our results suggest that the driving mechanisms for the variation of bacterial assemblages differ in different habitats or relative abundances of taxa. The bacterial communities of thermokarst lakes, especially rare subcommunities or particularly in sediment, might be strongly structured by environmental filtering and geographical isolation, potentially driving these assemblages that tend to be more compositionally distinct.

In addition, it has been widely studied that the community assembly processes can influence biogeochemical function by impacting community membership (Bier et al., 2015; Graham et al., 2016a; Graham and Stegen, 2017; Le Moigne et al., 2020; Luan et al., 2020). The relative contribution of deterministic and stochastic processes to community structuring is hypothesized to alter biogeochemical function (Strickland et al., 2009; Nemergut et al., 2013; Pholchan et al., 2013; Graham and Stegen, 2017). However, many recent studies suggest that the community assembly processes influence the biogeochemical function in uncertain ways (Wallenstein and Hall, 2012; Rocca et al., 2015; Graham et al., 2016b). For example, dispersal can suppress biogeochemical function due to the increasing proportion of maladapted taxa, which may invest more energy and resources in cell maintenance to survive rather than cellular machinery to drive biogeochemical processes (Strickland et al., 2009; Nemergut et al., 2013; Graham and Stegen, 2017). On the contrary, the “portfolio effect” suggests that dispersal enhances community functions because high diversity communities might occupy more niche spaces to reduce direct competition and contain more beneficial traits than lower diversity communities (Tilman et al., 1998; Schindler et al., 2010). Moreover, aside from the positive influences of selective biogeochemical function due to intensifying metabolic capabilities of the locally adapted taxa, selection may also impede biogeochemical function by eliminating the members mediating scarce resource metabolism (Loreau and Hector, 2001; Knelman and Nemergut, 2014). Although the relationships between community assembly and biogeochemical function are inconsistent according to previous studies, it is important to include the community assembly mechanisms in the understanding of microbial-mediated biogeochemical processes (Bier et al., 2015; Leibold et al., 2017; Mori et al., 2018). The larger the variation of the relative contributions of dispersal vs. selection, the stronger the influence of assembly processes on biogeochemical function (Graham and Stegen, 2017). In our study, the biogeochemical processes were not quantified. However, according to our results, we hypothesized that the high turnover rate of the whole bacterial communities in sediment and water might suggest that species experiencing replacement adapt to local environments and may have positive influences on the biogeochemical function. The low turnover rate of abundant subcommunities and high turnover rate of rare subcommunities suggest that the assembly processes of abundant and rare subcommunities might have opposite influences on the biogeochemical processes of the thermokarst lakes. Although our data cannot verify the hypotheses mentioned earlier, it is thrilling and enlightening to unravel the influences of abundant and rare subcommunities’ assembly on biogeochemical cycling in these lakes.

Integrating the differences in taxonomic composition and β-diversity patterns of bacterial communities in sediment and water in the whole, abundant, and rare communities/subcommunities, we can propose the hypothesis that the bacterial communities in water and sediment are initially originated from the same source (permafrost) and then diverged into two distinct communities due to the environmental divergence and the different assemblage rules in constructing communities. Further study of the bacterial community divergence during the thermokarst lakes’ formation and evolution processes would promote our understanding of the ecological consequences of future climate change.

## Conclusion

Thermokarst lakes are important and widespread aquatic ecosystems in cold regions and extremely vulnerable to accelerating climate change. Assessing the community composition and spatial pattern, as well as their underlying driving mechanisms in sediment and water, is pivotal in understanding the heterogeneous and fast-changing nature of thermokarst lake ecosystems. This study highlights the differences and linkages of sediment and water in physicochemical properties, taxonomical composition, and diversity patterns. Moreover, the underlying mechanisms driving taxa distributions patterns were also be revealed. Different β-diversity patterns existed between abundant and rare subcommunities and between sediment and water. This integral study of bacterial communities can enhance our understanding of the community assembly rules and ecosystem structures and processes of the thermokarst lakes.

## Data Availability Statement

The data presented in the study are deposited in the National Genomics Data Center (NGDC) repository, accession number CRA004269, PRJCA005279.

## Author Contributions

ZR: conceptualization, methodology, writing, visualization, investigation, and data analysis. CZ: methodology, writing, visualization, and data analysis. XL: writing- reviewing and editing, visualization. KM: writing- reviewing and editing, laboratory analyses. BC: conceptualization, writing- reviewing and editing, supervision. All authors contributed to the article and approved the submitted version.

## Conflict of Interest

The authors declare that the research was conducted in the absence of any commercial or financial relationships that could be construed as a potential conflict of interest.

## Publisher’s Note

All claims expressed in this article are solely those of the authors and do not necessarily represent those of their affiliated organizations, or those of the publisher, the editors and the reviewers. Any product that may be evaluated in this article, or claim that may be made by its manufacturer, is not guaranteed or endorsed by the publisher.
