# Supplementary material for: Abundant and Rare Bacterial Taxa Structuring Differently in Sediment and Water in Thermokarst Lakes in the Yellow River Source Area, Qinghai-Tibet Plateau
Source: Front Microbiol. 2022 Mar 29;13:774514. doi: 10.3389/fmicb.2022.774514 (PMC9002311; doi:10.3389/fmicb.2022.774514)
Supplement: Supplementary file 1 [file Data_Sheet_1.docx]

# Abundant and rare bacterialtaxa structuring differently in sediment and water inthermokarst lakes in the Yellow River Source area, Qinghai-Tibet Plateau

# Running Head: Bacterial structuring in thermokarst lakes

Ze Ren^1,2#^, Cheng Zhang^1,3#^, Xia Li^1,2*^,Kang Ma^2^, Baoshan Cui^1,2*^

1 Advanced Institute of Natural Sciences, Beijing Normal University, Zhuhai 519087, China

2 School of Environment, Beijing Normal University, Beijing 100875, China

3 School of Engineering Technology, Beijing Normal University, Zhuhai 519087, China

4 College of Arts and Sciences, Beijing Normal University, Zhuhai 519087, China

**#Authors contributed equally**

***Corresponding Author**:

Xia Li,[lixiabnu@bnu.edu.cn](http://lixiabnu@bnu.edu.cn), Advanced Institute of Natural Sciences, Beijing Normal University, Zhuhai 519087, China

Baoshan Cui, [cuibs@bnu.edu.cn](mailto:cuibs@bnu.edu.cn), School of Environment, Beijing Normal University, Beijing 100875, China

## Supplementary Information

Figure S1 Rarefaction curve of operational taxonomic units (OTUs) in each sample site at 97% nucleotide sequence identity threshold. (a) sediment samples. (b) water samples.


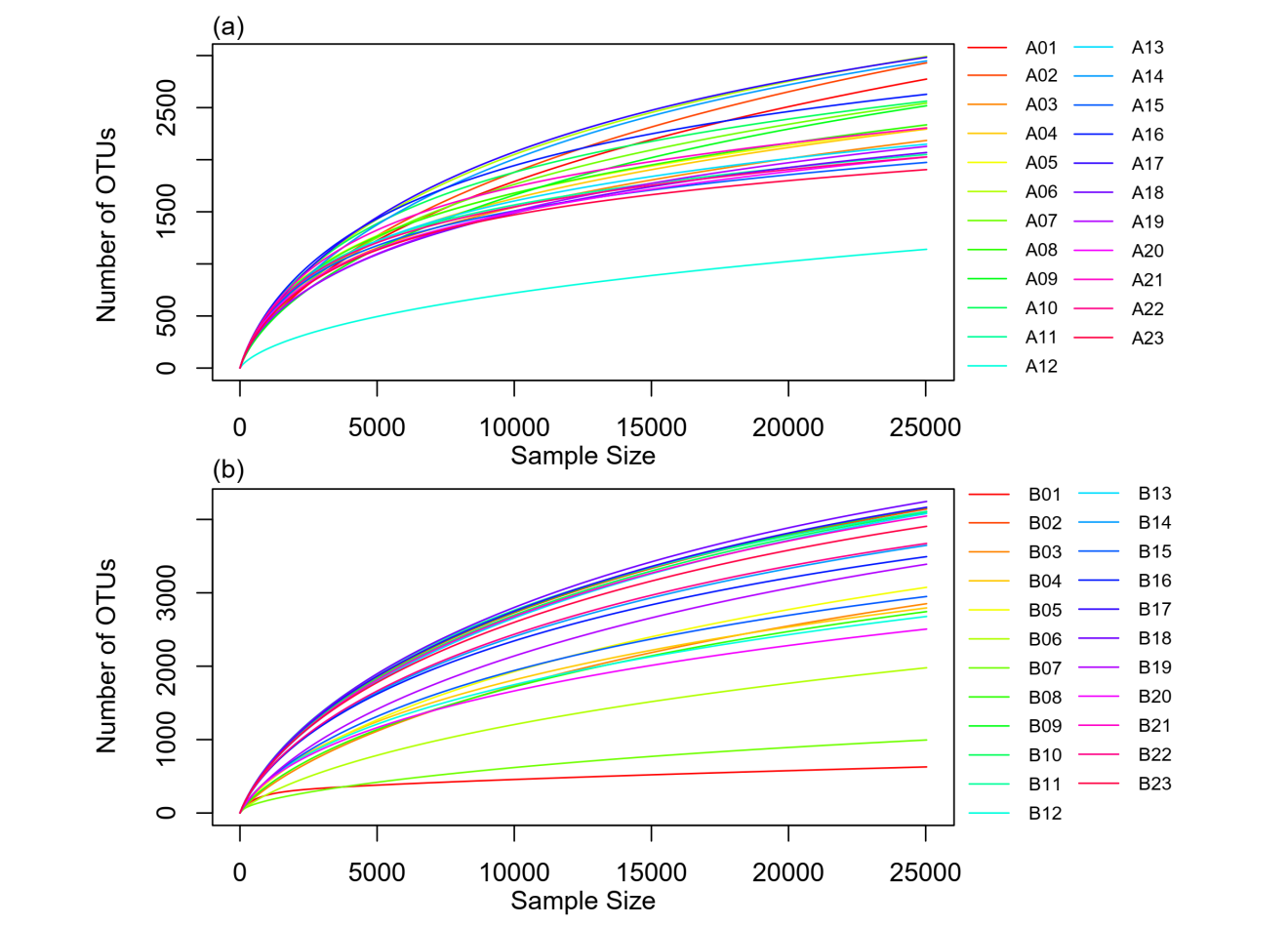


Figure S2The proportion of the richness and relative abundance of OTUs in abundant and rare subcommunities compared to the whole bacterial communities in each sample.


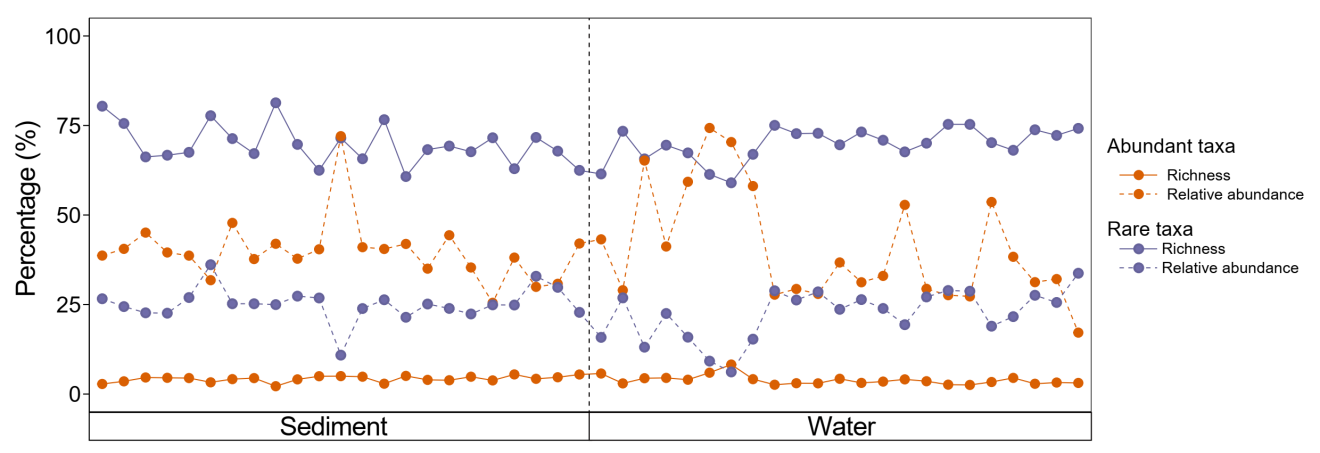


Figure S3The comparison of beta-partitioning ratios between whole, abundant, and rare taxa for paired sites of only sediment samples, between sediment and water samples, and only water samples, respectively. (a) Contribution of turnover component (βturn) to total β-diversity calculated as the Sorensen dissimilarity (βsor). (b) Contribution of nestedness‐resultant fraction (βnest) to total β-diversity calculated as the Sorensen dissimilarity (βsor). The different low-case lettersrepresent significant differences of the mean values using ANOVA.


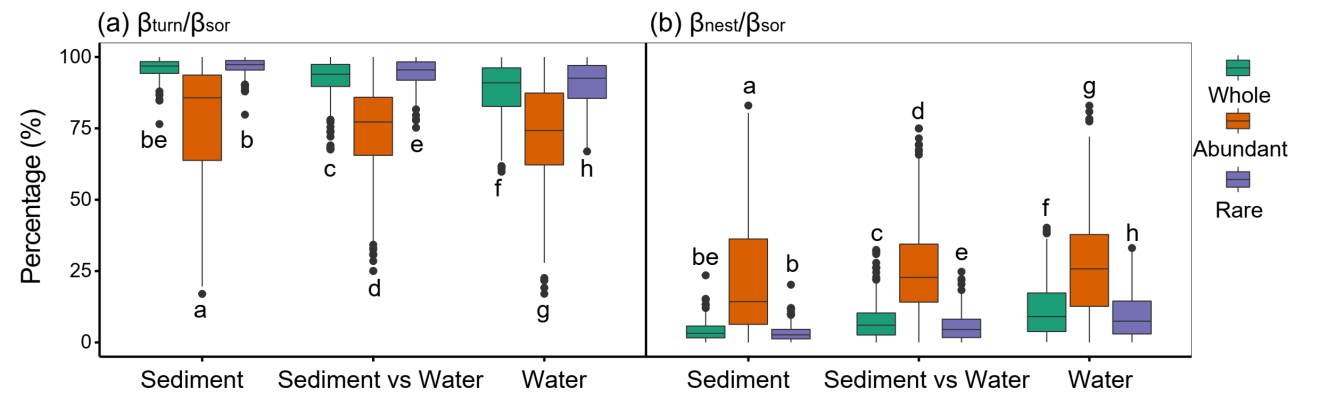


Figure S4 Top 25 OTUs in sediment and water in the thermokarst lakes


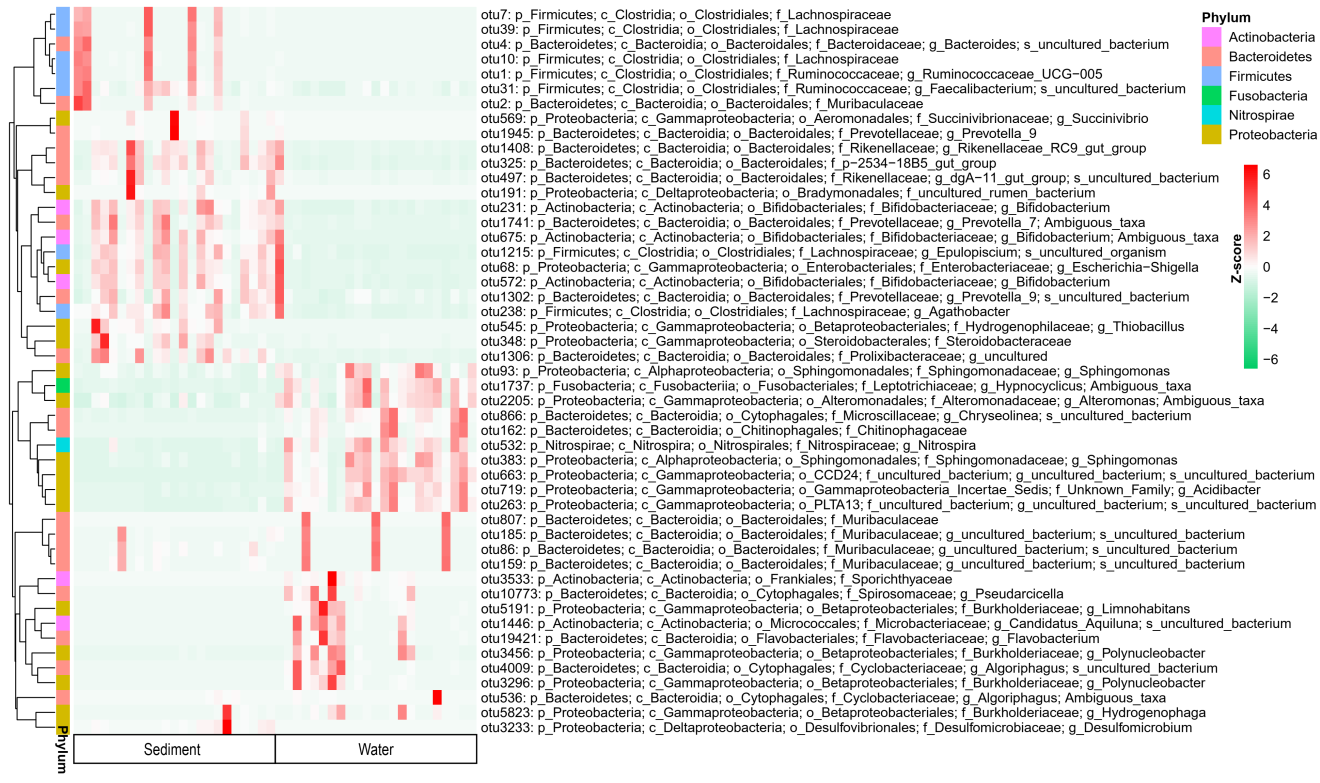


Table S1 The basic physicochemical properties of sediment and water samples.

|  | Minimum | Maximum | Mean | Standard Deviation | Coefficient Variation (%) |
| --- | --- | --- | --- | --- | --- |
| Sediment |  |  |  |  |  |
| pH | 8.835 | 9.835 | 9.354 | 0.246 | 2.631 |
| Conductivity (us/cm) | 204 | 1857 | 513 | 382 | 74 |
| SOC (g/kg) | 4.672 | 47.236 | 14.620 | 10.367 | 70.906 |
| TN (g/kg) | 0.280 | 2.550 | 1.092 | 0.645 | 59.045 |
| TP (g/kg) | 0.151 | 0.562 | 0.372 | 0.085 | 22.852 |
| Water |  |  |  |  |  |
| pH | 7.580 | 9.670 | 8.803 | 0.515 | 5.856 |
| Conductivity (us/cm) | 312 | 4849 | 1732 | 1416 | 82 |
| DOC (mg/L) | 1.590 | 43.850 | 16.477 | 10.438 | 63.347 |
| TN (mg/L) | 0.181 | 1.099 | 0.607 | 0.214 | 35.260 |
| TP (mg/L) | 0.039 | 0.162 | 0.100 | 0.028 | 27.871 |

Table S2 The differences between bacterial communities in sediment and water tested by analyses of Adonis, Anosim, and MRPP.

| Bacterial communities | Adonis | |  | Anosim | |  | MRPP | |
| --- | --- | --- | --- | --- | --- | --- | --- | --- |
|  | R^2^ | P |  | R | P |  | δ | P |
| Whole community | 0.192 | 0.001 |  | 0.662 | 0.001 |  | 0.847 | 0.001 |
| Abundant subcommunity | 0.241 | 0.001 |  | 0.661 | 0.001 |  | 0.805 | 0.001 |
| Rare subcommunity | 0.172 | 0.001 |  | 0.73 | 0.001 |  | 0.889 | 0.001 |
